# Caregivers’ knowledge, attitudes, and oral health practices at long-term care facilities in KwaZulu-Natal

**DOI:** 10.4102/hsag.v28i0.2147

**Published:** 2023-03-03

**Authors:** Sonam Balwanth, Shenuka Singh

**Affiliations:** 1 Discipline of Dentistry, School of Health Sciences, University of KwaZulu-Natal, Durban, South Africa

**Keywords:** caregivers, long-term care, institutionalised residents, oral health care, oral hygiene, oral health knowledge, oral health practices

## Abstract

**Background:**

Vulnerable individuals residing at long-term care facilities require special oral health consideration. Examining concepts of oral health and hygiene practices of caregivers becomes essential for understanding the quality of oral health services provided to residents.

**Aim:**

This study explored the oral health–related knowledge, attitudes and practices (KAP) of caregivers.

**Setting:**

Long-term care facilities in the eThekwini District, KwaZulu-Natal.

**Methods:**

A cross-sectional study was conducted at seven long-term care facilities among 188 caregivers who completed a self-administered questionnaire. Data were collated and analysed using the Statistical Package for the Social Sciences (SPSS) version 24. Inferential techniques included an analysis of variance (ANOVA) test. A *p*-value ≤ 0.05 was considered to be statistically significant.

**Results:**

Participants reported that dentures do not to be cleaned (*n* = 139; 73.9%). Participants (*n* = 70; 37.2%) reported that some medications have oral side effects. Most participants (*n* = 173; 92%) were optimistic about improving their oral health knowledge and skills. Participants (*n* = 108; 57.4%) only reported flossing when they had food trapped between their teeth. Few participants (*n* = 30; 16%) reported visiting the dentist every 6 months.

**Conclusion:**

Participants had a positive attitude to improve their oral health-related knowledge and practices. However, the study showed that there is a need to scale-up oral health education and training activities for caregivers.

**Contribution:**

It is envisaged that findings of this study will demonstrate the importance of oral health-related knowledge among caregivers in providing better oral health care through improved attitudes and practices.

## Introduction

Institutionalised residents have special oral health needs and requirements, but their oral health remains a neglected aspect of health care (Shah et al. 2018). The frail and elderly, young children and individuals with physical and learning disabilities residing at long-term care facilities constitute a marginalised and vulnerable population (Abullais et al. 2020; Miranda et al. 2020; Shah et al. 2018; Stancic et al. 2016). Oral disease, especially among institutionalised residents, is rife and therefore maintenance of their oral health is important in ensuring ‘good quality of life and overall well-being’ (Gil-Montoya et al. 2015; Shah et al. 2018). However, physical and mental limitations, emotional stress and chronic illness have a significant impact on their ability to perform adequate oral hygiene activities for themselves, resulting in unmet health needs (Abullais et al. 2020; Miranda et al. 2020; Stancic et al. 2016). A Serbian study found that the institutionalised elderly population were susceptible to dental caries, periodontal disease and tooth loss, especially among dependent and cognitively impaired residents (Stancic et al. 2016). Individuals with disabilities residing in long-term care facilities in Saudi Arabia were described as having compromised periodontal health (Abullais et al. 2020). Similarly, children living in orphanages in India showed a high prevalence of dental caries, gingivitis and dental trauma (Hans et al. 2014; Shah et al. 2016). Furthermore, inadequate oral hygiene and oral disease has been found to lead to nutritional deficiencies and systemic diseases in individuals, as in the case of bacterial endocarditis, pneumonia and pulmonary abscesses (Gil-Montoya et al. 2015; Stancic et al. 2016). It is evident that the prevalence of poor oral health among institutionalised residents is considerably high, and therefore, introspection into oral health care within long-term care facilities is necessary.

Caregivers thus play a vital role in providing preventive and promotional oral health care to residents by assisting and encouraging them to take better care of their oral hygiene (Miranda et al. 2020). Qualified nurses of long-term care facilities oversee the planning and evaluation of oral care; however, daily oral health care activities are predominantly performed by caregivers with varying levels of knowledge (Shah et al. 2018). Although oral health is integrated within the nursing curriculum, it is not always applied practically (Safi & Nasrallah 2017). Numerous international studies have found reasons for the unmet oral health needs of institutionalised residents (Eadie & Schou 1992; Petrovski et al. 2019; Simons et al. 2000). Petrovski et al. (2019) found that the high incidence of oral disease among institutionalised residents may be a result of an oversight in oral hygiene prioritisation among caregivers. Additionally, caregivers are often inundated with a high workload, resulting in time constraints to perform oral health care for residents (Eadie & Schou 1992). Previous studies found that the lack of oral health knowledge and training were significant limiting factors as to why caregivers do not perform proper oral health care for residents (Adams 1996; Eadie & Schou 1992; Khanagar 2014; Wardh et al. 2002). Furthermore, caregivers’ reluctance to perform oral health care for residents included respect for residents’ privacy, residents’ right to dignity and residents’ personal wishes to refuse oral care (Ettinger & Miller-Eldridge 1985; Weeks & Fiske 1994). These barriers hinder appropriate oral health care among institutionalised residents, and therefore, valuable solutions such as oral health education with residents and training of caregivers have been frequently recommended (Petrovski et al. 2019; Simons et al. 2000).

The knowledge and educational characteristics of caregivers at long-term care facilities are fundamental in providing optimal oral health care. Oral health education and training, as well as integration of oral health services into routine health care services, have been recommended as the most beneficial factor in improving oral health access to institutionalised residents (Petrovski et al. 2019; Thema & Singh 2013). There is limited relevant evidence internationally and a scarcity of data accessible in South Africa examining oral health care in long-term care facilities. Caregivers have direct influence on the effect of oral care of institutionalised residents; therefore, the objective of the study was to assess caregivers’ knowledge, attitudes and practices (KAP) regarding oral health.

## Methods

This cross-sectional, quantitative study was conducted from June 2021 to July 2021. Utilising a quantitative research method enabled the author to analyse statistical data, thereby removing biases from the research and producing more accurate findings because of the scientific and objective nature of the method (Eyisi 2016). The oral health–related KAP of caregivers were evaluated by means of a self-administered questionnaire.

The sample consisted of 188 purposely selected voluntary participants. Study sites were purposively selected from the ‘eThekwini Health and Well-being Service Provider Directory 2018’ and a website called ‘Senior Service Retirement Places’ on search engine company Google (eThekwini Municipality 2018; Senior service 2021). A total of seven long-term care facilities were selected for the study, which included six old age homes and one children’s home in the eThekwini district, all of which offer long-term care.

The research instrument composed of a self-administered questionnaire consisting of open- and closed-ended questions. The questionnaire was based on a previously developed questionnaire by Khanagar et al. (2014). The questionnaire was validated by means of a pilot study, which included five caregivers from a long-term care facility in eThekwini to pretest the efficiency of the questionnaire. These caregivers were excluded from the final questionnaire. Pretesting aided in identifying grammatical errors, clarifying any ambiguity in the questions posed and preventing misunderstanding or misinterpretation in the final questionnaire. The questionnaire comprised 30 items divided into three sections. The first section included questions on participants’ biographical information, such as gender, age, level of education, years of work experience and self-reported oral symptoms experienced, such as halitosis, toothache, oral abscesses, missing teeth, decayed teeth, bleeding gums, mouth sores, speech problems, difficulty chewing, digestion problems and unhappiness regarding their smile. The second section focused on participants’ knowledge, which consisted of open- and closed-ended questions based on defining dental terms, clinical identification of oral disease, as well as the cause and prevention of oral disease. Oral health–related knowledge questions on oral cancer and geriatric concepts were structured in the form of a Likert scale with the format of responses: (1) strongly agree, (2) agree, (3) not sure, (4) disagree and (5) strongly disagree. With regard to oral health practices, questions were centred on participants’ dental habits, frequency of dental visits, dietary habits and self-reported oral health habits and practices. The third section of the questionnaire included two items on attitude in the form of a Likert scale. These items elicited responses pertaining to the prioritisation of oral health practices and training among the participants, job satisfaction and barriers encountered in treating residents at long-term care facilities.

Completed data were placed in a sealed envelope by the matron or manager and collected by the author from each long-term care facility. Data were analysed using the Statistical Package for the Social Sciences (SPSS) version 24.0 (IBM Corporation, Armonk, New York, United States). Univariate descriptive statistics, such as frequency and mean distribution of categorical data, were conducted for all variables. The responses to the open-ended questions were grouped, and emergent themes were examined and compared for possible associations. Inferential techniques included an analysis of variance (ANOVA) test to assess the possible differences between the outcome variables (oral health KAP) and the independent variables (gender, age group, level of education, place of work and years of work experience). A *p* ≤ 0.05 was considered to be statistically significant. A Pearson correlation test was used to assess the association with the KAP scores.

### Ethical considerations

The study was granted ethical clearance by the Biomedical Research Ethics Committee at the University of KwaZulu-Natal (ref. no. BREC/00002633/2021). Participants were invited to participate in the study, and written informed consent was obtained. Study participants were informed that the study was voluntary and that participants were free to withdraw from the study at any stage, without any negative consequences. The questionnaire was administered in English after confirming that all the participants were comfortable with the language. All other ethical issues, such as confidentiality and anonymity, were maintained.

## Results

The majority of the participants were female (*n* = 182; 96.8%). Most of the participants (*n* = 52; 27.7%) were within the age group 30–36, followed by 45 participants (23.9%) within the age group 37–42. With regard to work experience, most of the participants (*n* = 92; 48.9%) had between 6 and 10 years of work experience. Almost all participants (*n* = 157; 83.5%) provided care to the elderly, followed by participants (*n* = 17; 9%) who provided care to adults with mental or physical impairments and participants (*n* = 14; 7.4%) who provided care to babies and children below 18 years old.

### Caregivers’ knowledge regarding oral health

The majority of the participants (*n* = 126; 67%) reported that the term ‘oral health’ meant preserving the health of the teeth and gums by means of brushing, flossing, irrigating and using other dental devices. Sixteen participants (*n* = 16; 8.5%) indicated that ‘oral health’ was the absence of disease, while some participants (*n* = 11; 5.9%) reported that ‘oral health’ was the prevention of oral disease. Few participants (*n* = 8; 4.3%) indicated that ‘oral health’ was an essential component of overall health and well-being. Six participants (*n* = 6; 3.2%) reported that ‘oral health’ was an oral condition, while 21 participants (11.2%) were unsure of the meaning of ‘oral health’. The majority of the participants (*n* = 125; 66.5%) had secondary-level education, while 63 participants (33.5%) had tertiary-level education. The ANOVA value indicated statistically significant differences in terms of participants’ knowledge (*p* = 0.007) as shown in Table 1. Participants with a tertiary level of education demonstrated higher knowledge (0.66 ± 0.3) when compared to those with a secondary level of education (0.55 ± 0.3).

**TABLE 1:** Relationship between demographic characteristics and knowledge, attitude and practice scores.

Variable	Number	Knowledge	*p*	Attitude	*p*	Practice	*p*
**Gender**			0.708		0.310		0.022**
Male	6	0.54 ± 0.3		0.57 ± 0.3		0.25 ± 0.1	
Female	182	0.59 ± 0.3		0.65 ± 0.2		0.37 ± 0.1	
**Age**			0.204		0.533		0.174
18–23 years old	1	0.00 ± 0.0		0.60 ± 0.0		0.40 ± 0.0	
24–29 years old	23	0.55 ± 0.4		0.60 ± 0.2		0.35 ± 0.1	
30–36 years old	52	0.55 ± 0.3		0.62 ± 0.2		0.37 ± 0.1	
37–42 years old	45	0.59 ± 0.3		0.64 ± 0.2		0.36 ± 0.1	
43–47 years old	31	0.66 ± 0.2		0.66 ± 0.2		0.36 ± 0.1	
48–53 years old	17	0.54 ± 0.3		0.66 ± 0.2		0.36 ± 0.1	
54–59 years old	16	0.61 ± 0.3		0.75 ± 0.2		0.44 ± 0.2	
60–66 years old	2	0.88 ± 0.1		0.70 ± 0.4		0.20 ± 0.0	
> 67 years old	1	0.83 ± 0.0		0.66 ± 0.0		0.60 ± 0.0	
**Education qualification**			0.007***		0.968		0.928
Secondary school	125	0.55 ± 0.3		0.65 ± 0.2		0.37 ± 0.1	
Tertiary education	63	0.66 ± 0.3		0.65 ± 0.2		0.37 ± 0.1	
**Work experience (years)**			0.745		0.544		0.481
1–5	52	0.60 ± 0.3		0.65 ± 0.2		0.39 ± 0.1	
6–10	92	0.57 ± 0.3		0.63 ± 0.2		0.36 ± 0.1	
11–15	27	0.56 ± 0.2		0.67 ± 0.2		0.39 ± 0.1	
> 15	17	0.64 ± 0.3		0.69 ± 0.2		0.35 ± 0.2	

Half of the participants (*n* = 94; 50%) indicated that the common causes of tooth decay were high sugar intake in the diet in combination with poor oral hygiene practices. Sixty-three participants (*n* = 63; 33.5%) reported that tooth decay was caused by poor diet alone (i.e. high sugar intake). Some participants (*n* = 15; 8%) were unsure about the common causes of tooth decay, while eight participants (4.3%) reported that the cause of tooth decay was multifactorial (poor diet, poor oral hygiene and causative bacteria).

The majority of participants reported that oral cancer can present in the throat (*n* = 137; 72.9%), gums (*n* = 122; 64.9%), tongue (*n* = 109; 58%) and floor of the mouth (*n* = 99; 52.7%). Fewer participants indicated that oral cancer can present on the roof of the mouth (*n* = 91; 48.4%), lips (*n* = 87; 46.3%) and the inner lining of the cheeks (*n* = 87; 46.3%).

The majority of participants (*n* = 149; 79.3%) reported that dentures must be removed at night-time (Table 2). Most of the participants indicated that the elderly and sickly with fragile gums may need to use a toothbrush with softer bristles (*n* = 141; 75%). Participants reported that dentures do need to be cleaned (*n* = 139; 73.9%). Some participants (*n* = 88; 46.8%) indicated that different types of toothpaste can sometimes cause mouth ulcers in the elderly and sickly, and other participants (*n* = 70; 37.2%) reported that some medications can cause swelling of the gums.

**TABLE 2:** Caregivers’ oral health–related knowledge.

Statement	Question	Oral health–related knowledge (*n* = 188)										
		Strongly agree		Agree		Unsure		Disagree		Strongly disagree		*p*
		*n*	%	*n*	%	*n*	%	*n*	%	*n*	%	
False teeth (dentures) must be removed at night-time	Q1	82	43.6	67	35.6	28	14.9	7	3.7	4	2.1	< 0.0001*
The elderly and sickly with fragile gums may need a toothbrush with softer bristles	Q2	50	26.6	91	48.4	36	19.1	5	2.7	6	3.2	< 0.0001*
False teeth (dentures) do not need to be cleaned	Q3	12	6.4	8	4.3	30	16.0	52	27.7	86	45.7	< 0.0001*
Different toothpastes can sometimes cause mouth ulcers in the elderly and sickly people I provide care to	Q4	21	11.2	67	35.6	80	42.6	14	7.4	6	3.2	< 0.0001*
Some medications can cause swelling of the gums of the people I provide care to	Q5	14	7.4	56	29.8	95	50.5	18	9.6	5	2.7	< 0.0001*

* , Statistically significant *p* < 0.05.

### Caregivers’ attitudes towards oral health care

The majority of participants reported feeling embarrassed and self-conscious if they had forgotten to brush their teeth (*n* = 174; 92.6%) (Table 3). Almost all participants (*n* = 173; 92%) indicated that they would like to gain more knowledge and skills about looking after their own oral health. The majority of participants (*n* = 150; 79.8%) felt that their oral health was as important to them as their overall general health. Conversely, 76.6% of the participants (*n* = 144) reported only visiting the dentist when they had experienced pain in their mouth, and 64.4% of the participants (*n* = 121) felt that visiting the dentist every 6 months was not one of their priorities. The majority of the participants reported performing correct oral hygiene procedures for themselves (*n* = 159; 84.6%). Most participants indicated that their oral hygiene habits and techniques could be improved (*n* = 154; 81.9%).

**TABLE 3:** Caregivers’ oral health–related attitudes.

Statement	Question	Oral health–related attitudes (*n* = 188)										
		Strongly agree		Agree		Unsure		Disagree		Strongly disagree		*p*
		*n*	%	*n*	%	*n*	%	*n*	%	*n*	%	
If I forget to brush my teeth, I feel embarrassed and self-conscious	Q1	94	50.0	80	42.6	5	2.7	3	1.6	6	3.2	< 0.0001*
I would like to gain more knowledge and skills about looking after my own oral health	Q2	79	42.0	94	50.0	8	4.3	4	2.1	3	1.6	< 0.0001*
My oral health is not as important to me as my overall general health	Q3	7	3.7	22	11.7	9	4.8	96	51.1	54	28.7	< 0.0001*
Pain in my gums and teeth is the only reason I visit the dentist	Q4	33	17.6	105	55.9	6	3.2	29	15.4	15	8.0	< 0.0001*
Visiting the dentist every 6 months is not one of my priorities	Q5	16	8.5	87	46.3	18	9.6	56	29.8	11	5.9	< 0.0001*
I feel that I am performing correct oral hygiene procedures for myself	Q6	44	23.4	115	61.2	22	11.7	7	3.7	0	0.0	< 0.0001*
I feel that my brushing and flossing habits and technique can be improved	Q7	41	21.8	113	60.1	22	11.7	11	5.9	1	0.5	< 0.0001*

* , Statistically significant *p* < 0.05.

Most of the caregivers (*n* = 126; 67%) reported being satisfied with their working hours at long-term care facilities. The majority of the participants indicated that caregiving is their passion, and they would like to work at long-term care facilities for a long time (*n* = 149; 79.2%), and almost all caregivers thoroughly enjoyed their jobs (*n* = 167; 88.8%).

Most of the caregivers reported that private-practice dentists are expensive and that is why they avoid going to the dentist for their regular check-ups (*n* = 129; 68.6%). The majority of the caregivers (*n* = 112; 59.6%) indicated that the cost of transport to the public dental department at hospitals was not a hindrance to visiting the dentist. Caregivers indicated that the long waiting times for dental care at the public hospitals was not a reason to avoid visiting the dentist (*n* = 109; 57.9%). Most caregivers reported that they have sufficient time to visit the dentist despite the demands of their job (*n* = 141; 75%). More than half of the participants (*n* = 97; 51.5%) reported that their financial commitments and cost of dental care did not prevent them from visiting the dentist.

The majority of the caregivers reported uncooperative behaviour as the main barrier to providing oral care to the residents (*n* = 98; 52.1%), followed by the lack of staff (*n* = 81; 43.1%), language barriers (*n* = 67; 35.7%), the lack of resources (*n* = 64; 34%) and the lack of time (*n* = 43; 22.9%).

### Caregivers’ oral health self-care practices

The ANOVA value measured for gender shows that while there were no statistically significant differences measured for attitude and knowledge (*p* > 0.05), a statistically significant difference was measured for practice (*p* = 0.022) (Table 1). The mean value measured for female respondents (0.37 ± 0.1) was higher when compared to those measured for the male respondents (0.25 ± 0.1). This suggests that female oral health practices were better than those of men in the study.

**FIGURE 1:**
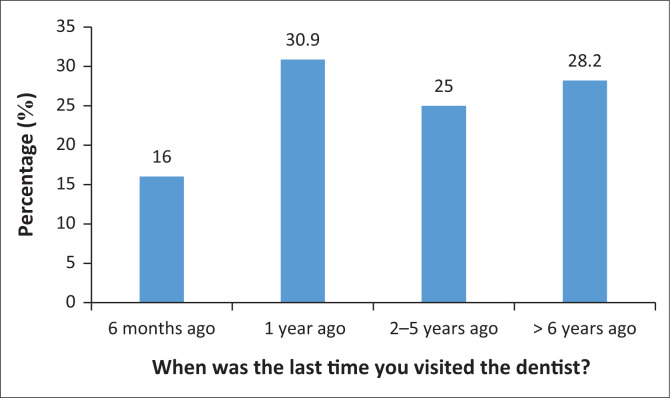
Frequency of caregivers’ dental visits.

**FIGURE 2:**
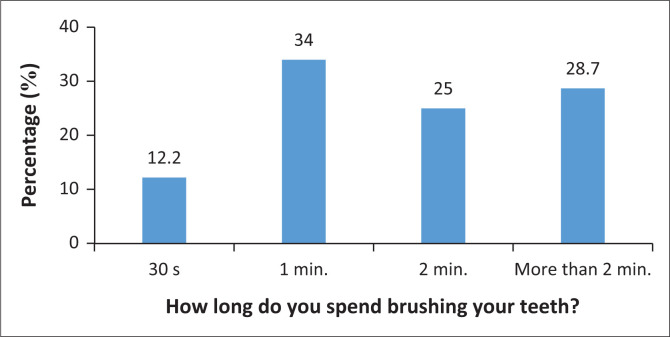
Time caregivers spend brushing their teeth.

Few participants (*n* = 30; 16%) reported visiting the dentist every 6 months (Figure 1). The majority of the participants (*n* = 101; 53.7%) reported cleaning their teeth with a toothbrush and toothpaste and no other dental aids. The majority of the participants (*n* = 137; 72.9%) indicated brushing their teeth twice a day. Few participants (*n* = 55; 29.3%) flossed daily, and many participants (*n* = 108; 57.4%) reported only flossing when they had food trapped between their teeth. The majority of participants (*n* = 98; 52.1%) reported using enough toothpaste to cover the entire toothbrush. Some participants (*n* = 64; 34%) reported spending 1 min brushing their teeth (Figure 2). Less than half of the participants (*n* = 90; 47.9%) indicated using mouthwash on a daily basis. Few participants (*n* = 77; 41%) reported brushing their teeth in a horizontal motion. The majority of the participants (*n* = 116; 61.7%) replaced their toothbrush every 1–3 months. Almost all participants (*n* = 186; 98.9%) reported not smoking.

**FIGURE 3:**
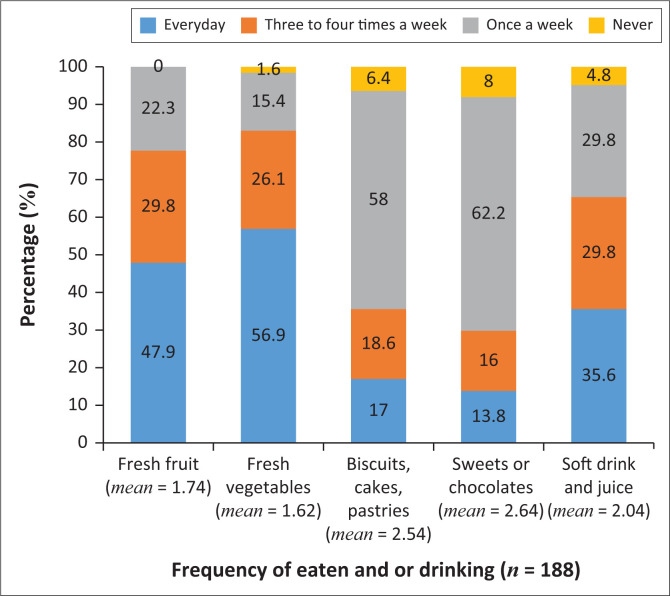
Caregivers’ dietary habits.

Most participants ate fresh vegetables (*n* = 107; 56.9%), and some participants ate fresh fruit (*n* = 90; 47.9%) on a daily basis (Figure 3). The majority of the participants ate sweets or chocolates (*n* = 117; 62.2%), biscuits and pastries (*n* = 109; 58%) once a week, while few participants (*n* = 67; 35.6%) drank soft drinks and juice on a daily basis.

The most commonly reported symptoms related to oral health experienced by participants included toothache (*n* = 111; 59%), tooth loss (*n* = 97; 51.6%), digestion problems (*n* = 88, 46.8%), decayed teeth (*n* = 75; 39.9%), bad breath (*n* = 72; 38.3%), bleeding gums (*n* = 69; 36.7%) and inability to chew or eat properly (*n* = 63; 33.5%). Less commonly reported symptoms included mouth sores (*n* = 53; 28.2%), oral abscess or swelling (*n* = 22; 22.3%), unhappiness regarding their smile (*n* = 32; 17%) and speech problems (*n* = 25; 13.3%).

More than half of the participants (*n* = 103; 54.8%) indicated visiting the dental department at a public hospital for dental treatment when they experienced pain or discomfort in their mouth. Some participants (*n* = 48; 25.5%) reported ingesting pain medication rather than visiting the dentist when they experienced oral pain. Few participants (*n* = 35; 18.6%) reported visiting the private dentist when they experienced oral pain, while 1% of the participants (*n* = 2) reported visiting the on-site dentist at their workplace when they experienced oral pain.

More than half of the participants (*n* = 103; 54.8%) indicated that the institutionalised residents are solely dependent on the caregiver to brush their teeth and look after their oral health. Less than half of the participants (*n* = 86; 45.7%) reported that the institutionalised residents are able to brush their own teeth under the supervision and assistance of the caregiver. Few participants (*n* = 7; 3.7%) indicated that the institutionalised residents are independent and can brush their own teeth.

### Correlation between oral health knowledge, attitude and practices

A Pearson correlation was computed to test the association in the KAP score. The correlation coefficient was interpreted using the following criteria: 0–0.25 = weak correlation, 0.25–0.5 = fair correlation, 0.5–0.75 = good correlation and greater than 0.75 = excellent correlation (Akoglu 2018).

The data shown in Table 4 revealed a fairly positive association between knowledge and attitude (*r* = 0.398, *p* < 0.001) and weak positive association between attitude and practice (*r* = 0.218, *p* = 0.003). No association was found between knowledge and practice (*p* > 0.05).

**TABLE 4:** Correlation between knowledge, attitude and practice scores.

Variable	Correlation coefficient	*p*
Knowledge–attitude	0.398**	< 0.001*
Knowledge–practice	0.092	0.208
Attitude–practice	0.218**	0.003*

* , Statistically significant *p* < 0.05.

** Pearson correlation significant at 0.01 level (two tailed).

## Discussion

This study investigated and provided valuable insight into the oral health–related KAP of caregivers, constituting the first such study to be conducted among caregivers in the eThekwini District, South Africa. The findings of this study indicated that caregivers’ educational qualifications influenced their oral health knowledge. No association was found between caregivers’ oral health knowledge and practice, which concurs with previous studies, postulating that improving one’s knowledge may be insufficient in bringing about behavioural change (Ashkanani & Al-Sane 2013). Other factors such as socio-economic and environmental factors play a significant role in influencing one’s behaviour to make better and more positive health choices and practices (Ashkanani & Al-Sane 2013; Behbehani & Scheutz 2004; Kay & Locker 1998; Vann et al. 2010). Caregivers’ awareness regarding the special oral health needs of the residents was inadequate. Despite caregivers expressing positive attitudes regarding oral health care and optimism in partaking in oral health training at long-term care facilities, barriers and challenges to the procurement of oral health care services for the residents were noted. Understanding these relevant aspects provides an opportunity to improve oral health provision to residents at long-term care facilities.

Caregivers with tertiary-level education exhibited better oral health knowledge than caregivers with secondary-level education. This finding concurs with previous studies conducted by Ashkanani and Al-Sane (2013) and Miranda et al. (2020), which showed that caregivers with a higher educational level demonstrated better oral health knowledge when compared with participants with lower educational levels. Abullais et al. (2020) found that caregivers who had a higher level of education exhibited more positive attitudes and a greater knowledge of oral health. Furthermore, a study conducted by Ashkanani and Al-Sane (2013) in Kuwait showed that the education and attitudes of caregivers were strongly associated with the level of knowledge. It is evident that caregivers with higher education levels exhibit better oral health–related knowledge and positive attitudes, which can possibly be attributed to their ability to understand and grasp concepts more efficiently than those with secondary-level education. Therefore, continual oral health education and training for caregivers is necessary, as it has the potential to improve their oral health knowledge, generate positive attitudes and improve their own oral hygiene habits, as well as those of the residents (Liu et al. 2017).

However, the majority of the caregivers in the current study had obtained secondary-level education (*n* = 125; 66.5%), while only 63 participants (33.5%) had tertiary-level education. Similarly, the majority of caregivers in a rehabilitation centre in Saudi Arabia had also obtained secondary-level education, with some caregivers not having any formal education at all (Shah et al. 2018). This finding calls for review in the selection criteria of caregivers, where careful consideration is placed on educational levels, to ensure uniformity in the standard of care provided to institutionalised residents. Moreover, better oral health training and education programmes have been frequently recommended to improve the knowledge among caregivers and quality of care to institutionalised residents (Petrovski et al. 2019; Shah et al. 2018; Stancic et al. 2016).

Oral health forms an important component of overall health and is the key indicator of the quality of life and well-being of an individual (World Health Organization [WHO] 2022). The majority of the caregivers in the study understood the term ‘oral health’ as being essential in the preservation of the oral cavity and the prevention of oral disease. However, despite caregivers’ adequate knowledge on the term ‘oral health’, the depth of their understanding of oral health or how to achieve it was insufficient, which was evident in the study’s findings regarding their own oral health practices.

Almost all caregivers (*n* = 157; 83.5%) reported providing care to the institutionalised elderly population. However, despite the majority of the participants reporting being aware of some aspects of the special oral health needs of older people, their reported knowledge was not optimal. A lack of adequate knowledge in the management of oral ailments can have dire consequences for the residents, negatively impacting their health and leading to poor quality of life (Gil-Montoya et al. 2015). Similarly, a study conducted by Sinavarat, Manosoontorn and Anunmana (2018) found that the majority of caregivers at elderly long-term care facilities did not realise that the use of some chronic medications among older people can make them susceptible to bacterial and fungal infections. On the other hand, a study conducted at a comprehensive rehabilitation centre for special-needs patients in Saudi Arabia showed that caregivers had adequate knowledge in addressing the oral health needs of their specific group of residents, thereby ensuring better oral health outcomes (Shah et al. 2018). Therefore, oral health training and education for caregivers forms an integral component in improving their knowledge and in turn enables caregivers to provide better care and address the special oral health needs of the residents (Shah et al. 2018).

Almost all caregivers (*n* = 173; 92%) indicated that they would like to improve their oral health knowledge and skills, and 79.8% (*n* = 150) of caregivers reported that their oral health is important and forms an essential aspect of their overall health. Similarly, a study conducted by Liu et al. (2017) in Taiwan investigating caregivers’ oral health knowledge, attitudes and behaviour towards children with disabilities at long-term care facilities showed that the majority of the caregivers demonstrated positive attitudes. In the current study, almost all caregivers expressed high job satisfaction in terms of working hours, passion and enjoyment. This finding is consistent with a previous study conducted in Switzerland among caregivers at nursing homes, which found that 86.6% of the caregivers were satisfied with their jobs (Schwendimann et al. 2016). Caregivers are often first-line health providers to the institutionalised residents, and thus the optimistic and positive attitudes expressed by the caregivers in the current study are encouraging for ensuring attendance and engagement in future oral health initiatives, education and training offered at long-term care facilities. Furthermore, caregivers have the ability to create oral health awareness at long-term care facilities and can therefore motivate residents under their care to improve on their oral health practices.

On the other hand, the majority of caregivers (*n* = 144; 76.6%) reported only visiting the dentist when they experienced dental pain. This finding raises a major concern regarding the lack of preventive and promotional oral health measures placed within the communities of caregivers. Conduction of oral screenings has been shown to be of great value in ensuring timeous detection of dental caries and in turn reducing oral morbidity and the need for invasive dental treatment such as dental extractions (Greenberg et al. 2010; Rebich et al. 1982). Similar sentiments were shared among caregivers in studies conducted by Liu et al. (2017) and Shah et al. (2018). Some caregivers in the current study (*n* = 121; 64.4%) felt that visiting the dentist every 6 months was not one of their priorities. Considering that the majority of caregivers in the current study (*n* = 111; 59%) reported experiencing toothache, the infrequency of dental visits among caregivers highlights issues of accessibility to oral health care and the lack of oral health prioritisation. Caregivers often serve as role models for residents under their care and ought to exemplify good oral health behaviours.

Caregivers reported uncooperative behaviour from the residents (*n* = 98; 52.1%) as the main barrier to providing oral care, which is consistent with previous studies (Abullais et al. 2020; Forsell et al. 2011; Willumsen et al. 2011). Stancic et al. (2016), on the other hand, found that a lack of time (*n* = 23; 39.7%), closely followed by a lack of cooperation from residents (*n* = 19; 32.8%), were the main barriers to providing oral care to the residents. Earlier studies have reported additional barriers to providing oral health care to residents, such as a lack of prioritisation, dislike for the task, resistance from residents and a lack of time or availability of resources (Frenkel, Harvey & Needs 2002; Kambhu & Levy 1993; Stein & Aalboe 2015; Weeks & Fiske 1994). Considering the physical and mental vulnerability of institutionalised residents and the reliance on caregivers for oral health care, the presence of these barriers implies that oral disease will contine to exacerbate within long-term care facilities. Furthermore, the current study found that the main barrier to caregivers obtaining personal oral health care was the high cost of dental treatment at private-practice dentists. This finding concurs with previous international studies about oral health care among caregivers (Holmavuo et al. 2022). Therefore, oral health prevention needs to be emphasised in the form of educational programmes for caregivers and residents at long-term care facilites to prioritise oral care duties for caregivers, which will encourage them to form good self-care oral health habits.

Caregivers demonstrated adequate oral health practices in terms of frequency of tooth brushing, as 72.9% of participants (*n* = 137) indicated brushing their teeth twice a day. Tooth brushing for at least 2 min a day has been shown to be effective in plaque control and aid in the prevention of oral diseases (Ganss et al. 2009; Steele & Walls 1997). However, only 25% of participants (*n* = 47) reported spending the recommended 2 min brushing their teeth, and only 29.3% of participants (*n* = 55) flossed daily. These findings are similar to a study conducted by Abullais et al. (2020), which found that only 55% of caregivers grasped the concept of performing correct oral hygiene procedures for themselves. Interestingly, almost all caregivers in the current study indicated performing correct oral hygiene procedures for themselves (*n* = 159; 84.6%) but also reported that their brushing and flossing habits and techniques could be improved (*n* = 154; 81.9%). Despite the results indicating no association between knowledge and oral health practices, research shows that good knowledge encourages a positive attitude that has the potential to lead to better oral health practices (Liu et al. 2017).

A well-balanced diet low in sugars and the cessation of tobacco usage has been recommended by the WHO for the prevention of oral disease and promotion of oral health (WHO 2022). Most caregivers in the study reported eating fresh vegetables (*n* = 107; 56.9%) and fresh fruits (*n* = 90; 47.9%) on a daily basis, while the majority of the caregivers reported eating more cariogenic foods such as sweets and chocolates (*n* = 117; 62.2%), biscuits and pastries (*n* = 109; 58%) once a week. Additionally, almost all participants (*n* = 186; 98.9%) reported not smoking. As perceived role models for the residents, caregivers should practise and maintain good dietary habits. Enforcing these habits is essential for reducing the risk of oral disease, as residents are more susceptible to developing nutritional deficiencies (Shah et al. 2018).

The study revealed that more than half of the caregivers (*n* = 103; 54.8%) reported being solely responsible for providing oral hygiene care for the residents. Numerous international studies have demonstrated that oral health education and training for caregivers at long-term care facilities lead to better oral health behaviours and in turn better oral health outcomes (Nicol et al. 2005; Peltola, Vehkalahti & Simoila 2007). Therefore, there is a dire need for stakeholders of long-term care facilities to routinely organise oral health education and training for caregiving staff. Frenkel, Harvey and Needs (2002) and Reigle and Holm (2016) recommended reinforcement and continual oral health education and training to help improve and keep caregivers’ knowledge and skills up to date. Petrovski et al. (2019) advocated for the creation of an oral health education protocol for caregivers and deemed it a beneficial way of preventing oral diseases among residents at long-term care facilities. Caregivers are more likely to exhibit better oral hygiene practices when their knowledge is optimal and attitudes are positive (Liu et al. 2017). Inclusion of oral health education and practical oral health activities within in-service training sessions should be conducted with prioritisation and reinforcement to bridge the gap between oral health–related knowledge and practice. Nurses play an important role in providing continual oral health guidelines and competency training for caregiving staff. The nursing curriculum should include more pertinent oral health topics based on the special oral health needs of the residents and be taught by well-versed dental professionals. Adopting a sustainable multidisciplinary approach can provide great support to the residents of long-term care facilities by incorporating routine oral screenings into other disciplines of health care. Preventive and promotional oral health activities for institutionalised residents are a valuable way of creating oral health awareness within long-term care facilities and encouraging caregivers to be more cognisant of oral health.

## Study limitations

This study provided important information on oral health–related KAP of caregivers. However, some limitations were noted, such as possible recall bias among the caregivers. The data were retrieved from self-reported questionnaires; therefore, by reason of social desirability, caregivers may not have demonstrated the current situation, which may have resulted in over- or under-reporting (Ahamed et al. 2015; Singh & Pottapinjara 2017). Because of the adherence to strict coronavirus disease 2019 (COVID-19) protocols, the number of long-term care facilities in eThekwini participating in the study was limited to seven. The study was also limited to eThekwini District; therefore, the findings cannot be generalised to other regions of KwaZulu-Natal or other regions in South Africa. Further research on a larger scale is required to explore the oral health–related KAP of caregivers at long-term care facilities in South Africa.

## Conclusion

Despite caregivers exhibiting positive and optimistic attitudes about caregiving and a willingness to gain more oral health knowledge and skills, the study demonstrated that caregivers require guidance and proper protocols on oral health care. This can be delivered through regular oral health educational and training programmes that emphasise the importance of oral hygiene for residents with special oral health needs.

## References

[CIT0001] Abullais, S.S., Al-Shahrani, F.M.F., Al-Gafel, K.M.S., Saeed, A.A., Al-Mathami, S.A., Bhavikatti, S.K. et al., 2020, ‘The knowledge, attitude and practices of the caregivers about oral health care at centers for intellectually disabled, in southern region of Saudi Arabia’, *Healthcare (Basel)* 8(4), 416. 10.3390/healthcare8040416 33096596 PMC7712856

[CIT0002] Adams, R., 1996, ‘Qualified nurses lack adequate knowledge related to oral health, resulting in inadequate oral care of patients on medical wards’, *Journal of Advanced Nursing* 24(3), 552–560. 10.1046/j.1365-2648.1996.22416.x 8876416

[CIT0003] Ahamed, S., Moyin, S., Punathil, S., Patil, N.A., Kale, V.T. & Pawar, G., 2015, ‘Evaluation of the oral health knowledge, attitude and behavior of the preclinical and clinical dental students’, *Journal of International Oral Health* 7(6), 65–70. PMC4479777 26124603

[CIT0004] Akoglu, H., 2018, ‘User’s guide to correlation coefficients’, *Turkish Journal of Emergency Medicine* 18(3), 91–93. 10.1016/j.tjem.2018.08.001 30191186 PMC6107969

[CIT0005] Ashkanani, F. & Al-Sane, M., 2013, ‘Knowledge, attitudes and practices of caregivers in relation to oral health of preschool children’, *Medical Principles and Practice* 22(2), 167–172. 10.1159/000341764 22986905 PMC5586720

[CIT0006] Behbehani, J. & Scheutz, F., 2004, ‘Oral health in Kuwait’, *International Dental Journal* 54(1), 401–408. 10.1111/j.1875-595x.2004.tb00018.x 15631104

[CIT0007] Eadie, D. & Schou, L., 1992, ‘An exploratory study of barriers to promoting oral hygiene through carers of elderly people’, *Community Dental Health* 9(4), 343–348. 1486522

[CIT0008] eThekwini Municipality, 2018, *eThekwini municipality health and wellbeing service provider directory 2018*, viewed 03 July 2020, from http://www.durban.gov.za/Documents/HWDirectory2018.pdf.

[CIT0009] Ettinger, R. & Miller-Eldridge, J., 1985, ‘An evaluation of dental programs and delivery systems for elderly isolated populations’, *Gerodontics* 1(2), 91–97. 3859447

[CIT0010] Eyisi, D., 2016, ‘The usefulness of qualitative and quantitative approaches and methods in researching problem-solving ability in science education curriculum’, *Journal of Education and Practice* 7(15), 94.

[CIT0011] Forsell, M., Sjögren, P., Kullberg, E., Johansson, O., Wedel, P., Herbst, B. et al., 2011, ‘Attitudes and perceptions towards oral hygiene tasks among geriatric nursing home staff’, *International Journal of Dental Hygiene* 9(3), 199–203. 10.1111/j.1601-5037.2010.00477.x 21356019

[CIT0012] Frenkel, H., Harvey, I. & Needs, K., 2002, ‘Oral health care education and its effect on caregivers’ knowledge and attitudes: A randomised controlled trial’, *Community Dental Oral Epidemiology* 30(2), 91–100. 10.1034/j.1600-0528.2002.300202.x 12000349

[CIT0013] Ganss, C., Schlueter, N., Preiss, S. & Klimek, J., 2009, ‘Tooth brushing habits in uninstructed adults-frequency, technique, duration and force’, *Clinical Oral Investigation* 13(2), 203–208. 10.1007/s00784-008-0230-8 18853203

[CIT0014] Gil-Montoya, J.A., De Mello, A.L., Barrios, R., Gonzalez-Moles, M.A. & Bravo, M., 2015, ‘Oral health in the elderly patient and its impact on general well-being: A nonsystematic review’, *Clinical Interventions in Aging* 2015(10), 461–467. 10.2147/CIA.S54630 PMC4334280 25709420

[CIT0015] Greenberg, B.L., Glick, M., Frantsve-Hawley, J. & Kantor, M.L., 2010, ‘Dentists’ attitudes toward chairside screening for medical conditions’, *Journal of the American Dental Association* 141(1), 52–62. 10.14219/jada.archive.2010.0021 20045822

[CIT0016] Hans, R., Thomas, S., Dagli, R., Bhateja, G.A., Sharma, A. & Singh, A., 2014, ‘Oral health knowledge, attitude and practices of children and adolescents of orphanages in Jodhpur city Rajasthan, India’, *Journal of Clinical and Diagnostic Research* 8(10), 22–25. 10.7860/JCDR/2014/9026.4948 25478441 PMC4253259

[CIT0017] Holmavuo, K., Suominen, A.L., Lammintakanen, J., Nykänen, I., Välimäki, T., Koponen, S. et al., 2022, ‘Informal caregivers’ perceptions of oral care and their association with the use of oral health services: A cross-sectional study among informal caregivers and their care recipients’, *Clinical and Experimental Dental Research* 8(2), 589–599. 10.1002/cre2.552 35368149 PMC9033540

[CIT0018] Kambhu, P.P. & Levy, S., 1993, ‘Oral hygiene care levels in Iowa intermediate care facilities’, *Special Care in Dentistry* 13(5), 209–214. 10.1111/j.1754-4505.1993.tb01498.x 7716694

[CIT0019] Khanagar, S., Jathanna, V.R., Rajanna, V., Naik, S., Kini, V.P. & Reddy, S., 2014, ‘Knowledge, attitude and practices of caretakers in relation to oral health of institutionalised elderly in Bangalore city, India’, *International Journal of Preventive & Clinical Dental Research* 1(2), 14–18.

[CIT0020] Liu, H.Y., Chen, J.R., Hsiao, S.Y. & Huang, S.T., 2017, ‘Caregivers’ oral health knowledge, attitude and behavior toward their children with disabilities’, *Journal of Dental Sciences* 12(4), 388–395. 10.1016/j.jds.2017.05.003 30895080 PMC6395374

[CIT0021] Miranda, G.H.N., Fagundes, C.F., Da Costa e Silva, A.B., Davis, L.L., Raiol Dos Santos, M.A. & Lima, R.R., 2020, ‘Perception and practices of caregivers of childhood and youth shelters concerning oral health in the city of Belém, Brazil’, *O Mundo da Saúde* 44, 144–151. 10.15343/0104-7809.202044144151

[CIT0022] Nicol, R., Sweeney, M.P., McHugh, S. & Bagg, J., 2005, ‘Effectiveness of health care worker training on the oral health of elderly residents of nursing homes’, *Community Dentistry and Oral Epidemiology* 33(2), 115–124. 10.1111/j.1600-0528.2004.00212.x 15725174

[CIT0023] Peltola, P., Vehkalahti, M.M. & Simoila, R., 2007, ‘Effects of 11-month interventions on oral cleanliness among the long term hospitalized elderly’, *Gerodontology* 24(1), 14–21. 10.1111/j.1741-2358.2007.00147.x 17302926

[CIT0024] Petrovski, M., Terzieva-Petrovska, O., Kovecevska, I., Minovska, A., Ivanovski, K. & Papakoca, K., 2019, ‘Oral health education of staff in long-term care institutions’, *Balkan Journal of Dental Medicine* 23(2), 63–67. 10.2478/bjdm-2019-0012

[CIT0025] Rebich, T., Kumar, J., Brustman, B.A. & Green, E.L., 1982, ‘The need for dental health screening and referral programs’, *Journal of School Health* 52(1), 50–53. 10.1111/j.1746-1561.1982.tb02265.x 6915287

[CIT0026] Reigle, J.A. & Holm, K., 2016, ‘Knowledge of oral health of nursing staff caring for disadvantaged older people’, *Journal of Nursing Education and Practice* 6(1), 31–38. 10.5430/jnep.v6n1p31

[CIT0027] Safi, M.A. & Nasrallah, R., 2017, ‘Knowledge and attitude of oral health among caregivers in nursing homes for elderly in Ga-Rankuwa, South Africa: A cross-sectional study’, Bachelor thesis, Department of Oral Health Sciences, University of Jönköping, Jönköping.

[CIT0028] Schwendimann, R., Dhaini, S., Ausserhofer, D., Engberg, S. & Zúñiga, F., 2016, ‘Factors associated with high job satisfaction among care workers in Swiss nursing homes: A cross sectional survey study’, *BMC Nursing* 15, 37. 10.1186/s12912-016-0160-8 27274334 PMC4895903

[CIT0029] Senior Service, 2021, *Comprehensive directory of retirement facilities in South Africa*, viewed 18 May 2021, from https://www.seniorservice.co.za/advanced-search/?province=Kwa+Zulu+Natal&city=DURBAN+Greater+Durban&property_type=Old+Age+Homes&section=properties.

[CIT0030] Shah, A., Naseem, M., Khan, M.S., Asiri, F.Y.I., AlQarni, I., Gulzar, S. et al., 2018, ‘Oral health knowledge and attitude among caregivers of special needs patients at a comprehensive rehabilitation centre: An analytical study’, *Annali di stomatologia (Roma)* 8(3), 110–116. 10.11138/ads/2017.8.3.110 PMC5897091 29682223

[CIT0031] Shah, A.F., Tangade, P., Ravishankar, T.L., Tirth, A., Pal, S. & Batra, M., 2016, ‘Dental caries status of institutionalised orphan children from Jammu and Kashmir, India’, *International Journal of Clinical Pediatric Dentistry* 9(4), 364–371. 10.5005/jp-journals-10005-1392 28127170 PMC5233705

[CIT0032] Sinavarat, P., Manosoontorn, S. & Anunmana, C., 2018, ‘Knowledge, attitudes, and behaviour towards oral health among a group of staff caring for elderly people in long-term care facilities in Bangkok, Thailand’, *Medical Dental Journal* 38(1), 23–38.

[CIT0033] Singh, S. & Pottapinjara, S., 2017, ‘Dental undergraduate students’ knowledge, attitudes and practices in oral health self-care: A survey from a South African university’, *African Journal of Health Professions Education* 9(2), 83–87. 10.7196/AJHPE.2017.v9i2.800

[CIT0034] Stancic, I., Petrović, M., Popovac, A., Vasović, M. & Despotović, N., 2016, ‘Caregivers’ attitudes, knowledge and practices of oral care at nursing homes in Serbia’, *Vojnosanitetski Pregled* 73(7), 668–673. 10.2298/VSP141001065S 29314800

[CIT0035] Steele, J. & Walls, A., 1997, ‘Strategies to improve the quality of oral health care for frail and dependent older people’, *BMJ Quality & Safety* 6(3), 165–169. 10.1136/qshc.6.3.165 PMC1055479 10173775

[CIT0036] Stein, P. & Aalboe, J., 2015, ‘Dental care in the frail older adult: Special considerations and recommendations’, *Journal of the California Dental Association* 43(7), 363–368. 26819997

[CIT0037] Suominen, A.L., Helminen, S., Lahti, S., Vehkalahti, M.M., Knuuttila, M., Varsio, S. et al., 2017, ‘Use of oral health services in Finnish adults-results from the cross-sectional health 2000 and 2011 surveys’, *BMC Oral Health* 17(1), 1–13. 10.1186/s12903-017-0364-7 PMC5402661 28438160

[CIT0038] Thema, L.K. & Singh, S., 2013, ‘Integrated primary oral health services in South Africa: The role of the PHC nurse in providing oral health examination and education’, *African Journal of Primary Health Care and Family Medicine* 5(1), 1–4. 10.4102/phcfm.v5i1.413 PMC5566226 28828870

[CIT0039] Vann, W.J., Lee, J.Y., Baker, D. & Divaris, K., 2010, ‘Oral health literacy among female caregivers: Impact on the oral health outcome in early childhood’, *Journal of Dental Research* 89(12), 1395–1400. 10.1177/0022034510379601 20924067 PMC3123718

[CIT0040] Wardh, I., Berggren, U., Andersson, L. & Sörensen, S., 2002, ‘Assessments of oral health care in dependent older persons in nursing facilities’, *Acta Odontologica Scandinavica* 60(6), 330–336. 10.1080/000163502762667342 12512881

[CIT0041] Weeks, J. & Fiske, J., 1994, ‘Oral care of people with disability: A qualitative exploration of the views of nursing staff’, *Gerodontology* 11(1), 13–17. 10.1111/j.1741-2358.1994.tb00097.x 7713537

[CIT0042] Willumsen, T., Karlsen, L., Naess, R. & Bjørntvedt, S., 2011, ‘Are the barriers to good oral hygiene in nursing homes within the nurses or the patients?’, *Gerodontology* 29(2), 748–755. 10.1111/j.1741-2358.2011.00554.x 22023222

[CIT0043] World Health Organization (WHO), 2022, *Oral health*, viewed 07 June 2022, from https://www.who.int/news-room/fact-sheets/detail/oral-health.

